# Parliamentary Inquiry on the Medical Examination of Recruits

**Published:** 1917-07-21

**Authors:** 


### PARLIAMENTARY INQUIRY ON THE MEDICAL EXAMINATION OF RECRUITS.

### Some Lay Evidence.

The official medical witnesses who have appeared before the Select Committee at its later sittings have given a detailed account of the working of the Medical Boards in relation to the Army Command, and have made it evident that, in relation to the number of men dealt with, complaints and blunders have been relatively few in number. But they allow that mistakes have been made, and this in both directions. Naturally, these are most conspicuous where unsuitable men are sent into the Army. The competent man who is rejected is rarely inclined to challenge the decision. The Army doctor apparently has difficulty in believing that his civilian brother can, without instruction, realise that not all soldiers are called upon to fight. But he can realise that the soldier once in the Army is caught in the wheels of the machine, and that the limitations which attend his admission may cease to be effective under military pressure. If he doubted it before, the doctrine has now been fully expounded in his ears. Officer after officer tells the Committee that a man who can do any work in civil life can do the same work under Army conditions. A man the subject of heart disease would in C3, according to one of the witnesses, be "as safe as a church"; and from another source we learn that even the chronic dyspeptic may be "considered" by the military *cuisine*. There are two points which have been brought out in evidence and which have a close bearing on such claims. One of these is that a man's classification may be raised, and even raised promptly, on the decision merely of his commanding officer and one medical officer. Hence the civilian doctor who labels the man in a lower class is far from certain that the label will have any enduring efficacy. That certain men may improve under military training and discipline is beyond doubt, and provision must be made for this. But the improvement clearly ought to be certified by an authority not less important than the one which fixed the original classification. Bearing in the same direction is a clause in the instruction issued to the Medical Boards in his Command by Surgeon-General Bedford insisting on greater care in the admission of men into the lower groups. The clause reads: "It must be borne in mind that recruits below categories B1 and C1 are liable, under a certain age, to be posted to reserve battalions for physical training, squad drills, etc." Exactly; but what in these circumstances becomes of the man of C3 level, who, we are assured, would rest in this classification "safe as a church." We submit that the chance of a rapid higher-grading of the recruit by an authority of limited weight, and the uncertainty of what amount of physical strain may be imposed on men of low classification, are two influences which cannot escape the attention of the civilian practitioner, and enlightenment in these directions would be distinctly helpful. The rest of the medical evidence goes to show that by circulars issued from the War Office, directly or indirectly, a certain amount of pressure has been exercised on the Medical Boards; that in some instances the military medical president has, and apart from his civilian colleagues, claimed a commanding voice in the classification of the recruits; and that not all complaints have received an efficient measure of investigation. On the other hand, there is plain evidence that up and down the land doctors have sacrificed their personal interests to help forward the country's cause, and that they have given efficient and courteous service on the Medical Boards. For such service there is little recognition other than the message of a quiet conscience, while mistakes, actual or alleged, lend themselves to headlines, and are therefore welcome to those whose business is to provide this variety of mental stimulus.

There is special interest in the evidence given by the laymen who have appeared before the Committee, particularly by those who have had a large experience as members of one or other of the Tribunals. Here the individual issue with all its complications comes in an acute form and decision is imperative. Speaking after the experience of 3,000 cases, Sir Ryland Adkins, M.P., a member of the Northamptonshire Appeal Tribunal, said that the contradictions in successive classifications by different medical authorities had been very embarrassing to himself and his colleagues; and he added that the difficulty is increasing. His conclusion is that the doctors are overworked and have not adequate time to deal with difficulties which arise in individual cases. The result from the Tribunal point of view is a sense of uncertainty and want of confidence, and hence members feel naturally that they do not get the clear-cut medical information which is necessary for the discharge of their responsibilities. With regard to complaints of tearing up certificates and similar discourtesies he recognised some exaggeration, but he could not doubt there was real ground for them. What Sir Ryland Adkins specially emphasised was the congestion and pressure under which the Medical Boards had to work. At the Central Boards, he said, the congestion is so great that to refer a case there is equivalent to granting the man a period of exemption for some months. He thought the number of these Boards ought to be increased. On the raising of a man's classification after admission to the Army he recognised that such occurrences have a very bad effect on public opinion, and he urged that such a step should only be taken under the sanction of a Medical Board; nor did he appear quite satisfied that the recruit admitted as competent for some special variety of work was not at times put to other and very different work. Another witness, Mr. A. Richardson, M.P., chairman of the Law Society's Appeal Tribunal, gave instances of mutually contradictory medical classifications and of complaints made about the examining doctors; the latter he regarded as indicating a want of supervision of the Boards, and he urged that disciplinary measures ought to be enforced against the offenders when the grievance was shown to be well founded.

